# Scabies infestation might predispose surgical site infection: Case report

**DOI:** 10.1016/j.ijscr.2024.109747

**Published:** 2024-05-08

**Authors:** Ali Engin Daştan, Arman Vahabi, Volga Öztürk, Mehmet Alp Özmen, Erhan Coşkunol, Kemal Aktuğlu

**Affiliations:** a Ege University School of Medicine, Department of Orthopaedics and Traumatology, Turkey; b Turgutlu State Hospital, Department of Orthopaedics and Traumatology, Turkey

**Keywords:** Orthopedic infection, Surgical site infection, Implant related infection, Sarcoptes scabei

## Abstract

**Introduction and clinical importance:**

Human scabies is a contagious skin condition caused by the Sarcoptes scabiei mite, leading to skin damage and subsequent mechanical irritation from scratching. This impaired skin integrity predisposes individuals to skin infections. While the association between scabies and skin infections caused by *Staphylococcus aureus* and *Streptococcus pyogenes* is well-documented, there is limited literature on the risk of surgical site infections in such cases.

**Case presentation:**

This case report aims to explore this risk by presenting a case of surgical site infection caused by *Streptococcus pyogenes* following surgery for a complex elbow injury in a patient with scabies infestation.

**Discussion:**

Scabies infestation leads to direct spread of bacteria and contributes to bacterial infection. Furthermore, complement inhibition and dysbiosis induced by the scabies may facilitate the occurrence of these bacterial infections.

**Conclusion:**

Skin infections are frequently encountered in scabies infestations. Preferred incision should be evaluated meticulously before surgery. Further studies are needed to reach a definitive conclusion on this subject.

## Highlights

• Human scabies, is a contagious skin condition prevalent in regions with low socioeconomic status and crowded living conditions.
• Infections associated with streptococci in implants can lead to significant mortality and morbidity.
• Preferred incision should be evaluated meticulously before surgery regarding scabies tunnels.

## 1 Introduction

Human scabies, caused by the Sarcoptes scabiei mite, is a contagious skin condition prevalent in regions with low socioeconomic status, crowded living conditions, and following wars or natural disasters. It manifests as itchy lesions, disrupting skin integrity and predisposing individuals to skin infections [[1], [2], [3]]. The association between scabies and pyoderma, caused by *Staphylococcus aureus* and *Streptococcus pyogenes*, is well-established [4]. It has been suggested that complement inhibition by Sarcoptes scabiei may contribute to *Streptococcus pyogenes* infections [5,6].

While the association between scabies and skin lesions is widely recognized, there is a lack of literature addressing the potential risk of surgical site infections in individuals with scabies. Our objective in this case report is to discuss this risk by presenting a case of surgical site infection caused by *Streptococcus pyogenes* following surgery for a complex elbow injury in a patient with concurrent scabies infestation.

## 2 Case report

**Fig. 1:**
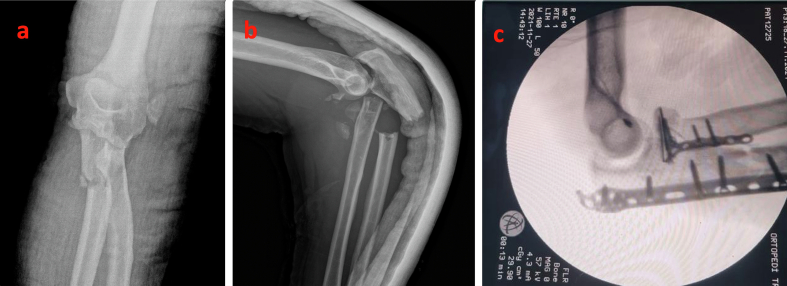
Elbow X-rays upon admission A) Anterior-posterior view. B) Lateral view. C) Intraoperative imaging.

This work has been reported in line with SCARE criteria [7]. A 42-year-old male patient presented to the emergency department .ED) with a complex elbow fracture following a fall from a height of 2 m. Physical examination did not reveal any open wounds, and neurovascular assessment was normal. Radiographic imaging showed a posterior fracture-dislocation of the elbow. A temporary closed reduction was performed in the ED, and the patient underwent surgery approximately 12 h after the injury, under axillary block anesthesia using a posterolateral Boyd approach. Operation time was 110 min. As antibiotic prophylaxis, 2 g of cefazolin were administered (Fig. 1).

After the operation, an articulated elbow orthosis was applied, and physiotherapy was initiated the following day. The allowed range of motion was gradually increased. Sutures were removed on the 14th day, and no wound problems were observed. However, the patient presented on the 40th day with complaints of pain, increased temperature, and swelling in his elbow. X-rays did not reveal any acute pathology. The patient had a body temperature of 37.9 °C and a white blood cell count of 27,800/μL. C-reactive protein (CRP) levels were elevated at 206.89 mg/l (reference range 0–5), and sedimentation was elevated at 46 mm/h (reference range < 15 mm/h). Aspiration was performed from the surgical area, yielding purulent fluid. Empirical antibiotic treatment with piperacillin-tazobactam and linezolid was initiated, and urgent debridement was performed under the diagnosis of peri-implant infection. The surgical site was opened along the same incision under axillary block anesthesia, and a large amount of purulent fluid was drained, which was associated with the elbow joint. Infected-looking soft tissues were debrided and thoroughly washed, while the implants were retained. Culture sampling was conducted, revealing *Streptococcus pyogenes* in the culture result. Consequently, the antibiotic treatment was modified to intravenous sulbactam-ampicillin on the second day following the culture results.

**Fig. 2:**
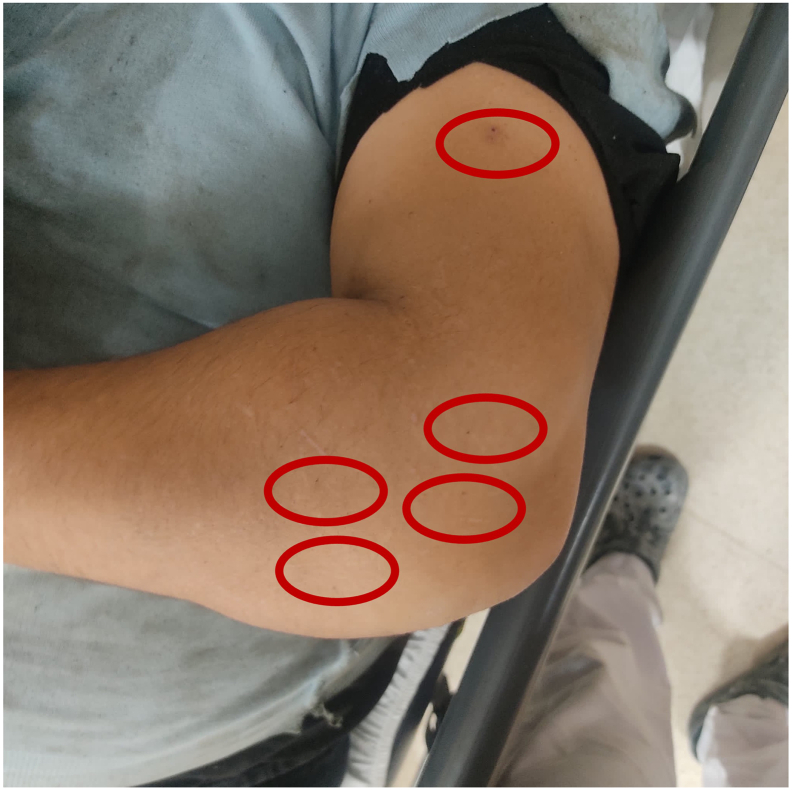
Photo of the elbow at first admission to emergency department. Note the black punctuations around the elbow (red circles). They thought to be remnant of the scabies tunnels.

The patient's medical history was further investigated upon the detection of *Streptococcus pyogenes*, a pathogen rarely encountered in orthopedic surgical site infections. Upon detailed inquiry, it was revealed that the patient had been diagnosed with scabies approximately one month prior and had received treatment for it. Initially, the patient had not disclosed this information, as his symptoms had subsided, and he feared that mentioning scabies would disrupt his ongoing treatment. However, upon reviewing photographs taken for documentation in the ED, lesions consistent with scabies tunnels were identified near the incision area (Fig. 2). Dermatology consultation was promptly sought, leading to the initiation of topical sulfur ointment. The use of incisional vacuum-assisted closure (VAC) was discontinued on the 7th day. As the patient's clinical and laboratory findings improved, intravenous antibiotic treatment was discontinued in the 6th week.

**Fig. 3:**
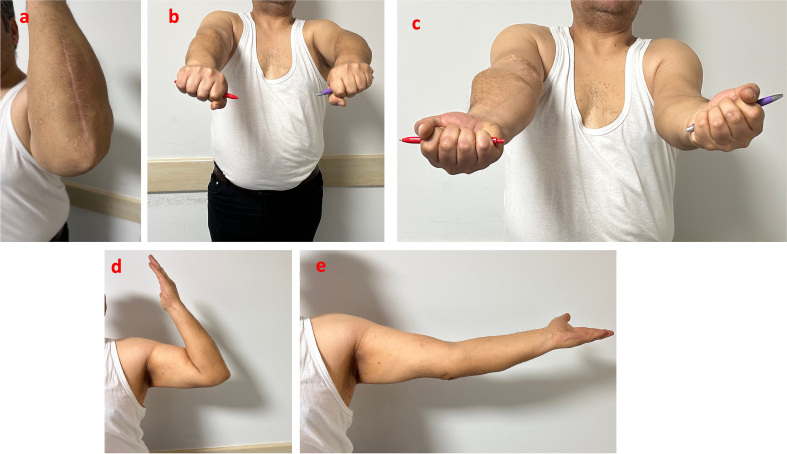
Status of surgical incision and elbow range of motion at postoperative one year. a) Surgical incision. b,c,d,e) Elbow range of motion.

No rigid restrictions were imposed on the patient's joint movement during the infection process. No recurrence of infection was observed during the one-year follow-up period. In the first postoperative year, there was a 20° limitation in extension, but a flexion of 130° was achieved. Although there was initially a 20° limitation in supination, full range of motion was eventually achieved in sıpination-pronation. Additionally, the patient achieved 5/5 motor strength in all muscle groups. (Fig. 3).

## 3 Discussion

Scabies, a contagious skin condition, has become a growing public health concern, with its prevalence increasing in recent years. Factors such as natural disasters, wartime conditions, and irregular migration contribute to its spread, particularly in settings with poor hygiene. Although traditionally associated with regions of lower socioeconomic status, scabies is observed globally, including in developed countries [8]. The condition disrupts skin integrity, predisposing individuals to bacterial infections. *Streptococcus pyogenes* and *Staphylococcus aureus* are commonly implicated bacteria in these infections [9]. The direct spread of these bacteria contributes to the prevalence of bacterial infections in scabies cases. Furthermore, complement inhibition induced by the scabies parasite may facilitate the occurrence of these bacterial infections [5,6,9,10]. In our case, the occurrence of a surgical site infection caused by *Streptococcus pyogenes*, an atypical pathogen for orthopedic infections, in a patient with no known risk factors for such infections prompted us to consider scabies infection as a predisposing factor.

Group A streptococcal (GAS) infections are known for their significant morbidity and mortality rates, particularly in cases of soft tissue infections. While in orthopedic practice GAS infections typically manifest as soft tissue infections, the risk of mortality is notably higher in individuals of advanced age, those with chronic diseases, and those who are immunocompromised [11]. In the case presented here, a *Streptococcus pyogenes* infection occurred following surgery for a complex elbow injury, which is unusual as this pathogen is not commonly associated with orthopedic surgical site infections. Therefore, we hypothesize that scabies infestation may have served as a predisposing factor in this uncommon etiology, especially considering that scabies lesions are frequently observed in the elbow area [12]. In this particular case, the surgical incision was made on the extensor side of the elbow, and initial photographic evidence revealed lesions consistent with scabies tunnels. We postulate that the compromised skin barrier in the area of these lesions may have facilitated the inoculation of *Streptococcus pyogenes*, leading to the subsequent infection.

Infections associated with streptococci in implants can lead to significant mortality and morbidity. The literature discusses the efficacy of debridement, antibiotics, and implant retention (DAIR) in managing streptococcus-associated prosthetic joint infections (PJI). Tamayo et al. concluded that DAIR is associated with a worse prognosis in PJI cases, [13] while Huotari et al. found that DAIR yields excellent results in cases of acute streptococcal PJI [14]. As observed in the current case, success can be achieved with early surgical debridement and implant retention coupled with appropriate antibiotic treatment.

The skin microbiome comprises various organisms, including bacteria, viruses, and fungi. Anatomical and physiological differences of the individual are the main reasons underlying the diversity in the skin microbiome [15]. Disruption of the skin flora has been linked to several skin conditions such as atopic dermatitis and psoriasis. Sarcoptes scabei, the causative agent of scabies, has been shown to induce dysbiosis in other species [16]. Considering the interaction between microbiota and the skin barrier, it could be speculated that the current infection in the patient may be associated with a Sarcoptes scabei infection [17]. Although conclusions cannot be definitively drawn from a single case, it may be prudent to consider making surgical incisions away from visible scabies tunnels or from typical tunnel sides in cases with active scabies infection. Additionally, in suspected cases, surgery could be postponed until scabies infestation is adequately controlled.

## 4 Conclusion

Skin infections are frequently encountered in scabies infestations. Preferred incision should be evaluated meticulously before surgery. Further studies are needed to reach a definitive conclusion on this subject.

## Ethical approval

No ethical board approval required for single case report. Informed consent received from patient regarding permission to use his data.

## Funding

No funding received for this report. There is no relationship with third parties.

## Author contribution

AED, AE, KA wrote the manuscript.

MAÖ, VÖ, AHÇ acquired the data.

EC and KA supervised the study.

## Guarantor

Dr. Arman Vahabi.

## Consent

Written informed consent was obtained from the patient for publication and any accompanying images. A copy of the written consent is available for review by the Editor-in-Chief of this journal on request.

## Conflict of interest statement

Nothing to declare.
